# Promising Short-Term Outcomes of Pulmonary Resections from Low-Volume Centre in Tier II City in India

**DOI:** 10.1007/s13193-024-02160-0

**Published:** 2025-01-09

**Authors:** Rajyalakshmi Puvvada, Rigved Nittala, Amita Sekhar Padhy, Pravallika Mekala

**Affiliations:** Department of Surgical Oncology, Homi Bhabha Cancer Hospital & Research Centre, Visakhapatnam, India

**Keywords:** Post-operative pulmonary complications (PPC), VATS, Air leak, Collapse, Low-volume centre

## Abstract

Lung cancer is one of the leading causes of cancer-related deaths in the world. Surgery offers the best potential cure, but as in any surgery, there are some complications that will be minimised with the improvement of surgical practice and treatment protocols. This study aims to analyse the short-term outcomes of thoracoscopic and open lung resections in a low volume centre at Visakhapatnam, India. Twelve patients who underwent thoracoscopic or open lung resections for primary and metastatic lung tumors from September 2022 to December 2023 at Homi Bhabha Cancer Hospital & Research Centre (HBCH&RC), Visakhapatnam, were analysed retrospectively. Data was collected from Electronic Medical Records including demographic data, neo-adjuvant and adjuvant therapy, surgical procedure performed, histopathology type, time to chest drain removal, length of hospital stay, perioperative mortality and morbidity occurring within 30 days after surgery. Statistical analysis was performed using SPSS version 26. A total 12 patients underwent pulmonary resections during the study period. Median age was 48.5 years. Surgical procedures performed were 4-bilobectomies, 7-lobectomies and 1-metastasectomy. Chest wall resection was performed for 2 cases, pericardial and diaphragm excision for one case. Surgical approaches were video-assisted thoracoscopic surgery (VATS), VATS converted to open and open—4 each. Major common complications were air leak and collapse which occurred in 33.3% of patients. Median duration of hospital stay was 6.5 days. There was no 30-day mortality. Safe outcomes are achievable after lung resections (both open and VATS procedures) in low-volume centre with trained and dedicated surgeons, anaesthesiologists and proper patient selection.

## Introduction

Lung cancer is the leading cause of cancer deaths [1]. Only one-fourth of cases in India are diagnosed in localised or locally advanced stage when they are treated by resection or combined modality treatment with or without surgery. The standard of care for stage I, II and selected cases of stage IIIA non-small cell lung cancer (NSCLC) is surgery followed by Adjuvant treatment as per final histopathology [2]. Lung resections are also performed for other primary lung tumours like carcinoids [3], Ewings sarcoma [4], metastasis [5], infections and trauma. Extent of lung resections range from non-anatomic wide excision, anatomical resections such as segmentectomy, lobectomy, to pneumonectomy. Surgery for early-stage lung cancer performed through VATS is associated with less pain, fewer in-hospital complications, leading to shorter length of hospital stay without any compromise to oncologic resection compared with open thoracotomy [6]. Post-operative pulmonary complications (PPCs) are associated with poor outcomes following lung resection [7]. Common PPCs are atelectasis, prolonged air leak, persistent pleural effusion, pneumonia and respiratory failure. Other complications include post-operative bleeding, surgical site infections (SSI), pulmonary embolism, systemic complications like cerebral infarction, MI and renal failure. Despite advances in anaesthesia and surgical care, PPCs still are a significant problem in modern practice. This study aims to analyse the introduction of thoracoscopic and open lung resections in a low-volume centre and to assess short term surgical outcomes.

## Materials and Methods

This was a retrospective study of all pulmonary resections for primary and metastatic lung tumours performed in Homi Bhabha Cancer Hospital & Research Centre (HBCH&RC) in Visakhapatnam from September 2022 to May 2024, focusing on both open and VATS procedures. A total 12 patients underwent pulmonary resections during this period. Data was retrieved from Electronic Medical Records, including information on demographic and clinical data: gender, age, smoking history, American society of anaesthesiology (ASA) score, neo-adjuvant & adjuvant therapy, perioperative data - consisting of surgical procedure, histopathologic type, time to intercostal tube drain (ICD) removal, length of hospital stay, operative morbidity and mortality occurring within 30 days after surgery.

All patients with primary non-small cell lung carcinoma were evaluated with PET CECT, MRI brain to rule out metastatic disease. For carcinoid tumours- DOTA PET; metastatic osteosarcoma- bone scan and CECT thorax; Ewings sarcoma & Synovial sarcoma—PET CECT, were performed as a part of metastatic work up. Biopsy either image guided or bronchoscope guided (if endo bronchial component) was performed in all cases. Functional evaluation was done with 6-min walk test and pulmonary function tests. Smokers were advised smoking cessation at least 4 weeks prior to surgery, but surgery was not delayed solely for this reason. Incentive spirometry was started early in pre-operative period. In our centre, invasive mediastinal staging was not performed prior to surgery due to lack of facilities. Mediastinal Staging for PET negative nodes was done by EBUS (outsourced).

All the surgeries were performed under general anaesthesia using double lumen intubation for single lung ventilation, with patient in lateral decubitus position. For open procedures, posterolateral thoracotomy incision was utilized. For VATS, Uniportal VATS technique was used. Main vessels and bronchus were divided with surgical stapler. For primary malignant primary lung tumours, systematic mediastinal lymph node dissection (SMLND) was performed. A chest drain was left in place for both open and thoracoscopic procedures. Post-operatively patients were mobilised within 24 h, urinary catheters if any & chest drains are removed early. Chest physiotherapy and incentive spirometry were started immediately once the patient was fully awake and continued or escalated as appropriate until resolution of pulmonary issues and usual mobility independence and exercise tolerance were restored. Adequate pain control in the post-operative period was achieved by thoracic epidural analgesia, systemic opioids and analgesics.

We analysed the 30-day post-operative morbidity and mortality retrospectively. All complications were graded using the Ottawa Thoracic Morbidity and Mortality classification defined in Table 1 [8]. Statistical analysis was performed using SPSS version 26.

**Table 1:** Ottawa thoracic morbidity and mortality classification

Grade	Definition
Minor	
Grade I	Any complication without need for pharmacologic treatment or other intervention
Grade II	Any complication that requires pharmacologic treatment or minor intervention only
Major	
Grade III	Any complication that requires surgical, radiologic, endoscopic intervention or multitherapy
Grade IIIa	Intervention does not require general anaesthesia
Grade IIIb	Intervention requires general anaesthesia
Grade IV	Any complication requiring intensive care unit management and life support
Grade IVa	Single organ dysfunction
Grade IVb	Multi-organ dysfunction
Death	
Grade V	Any complication leading to the death of the patient

## Results

- Demographic details: during the study period, 12 patients underwent pulmonary resection. Baseline demographics are shown in Table 2.

**Table 2:** Demographics (*n* = 12)

Year-wise number of cases	2022	2 (16.6%)
	2023	9 (75.0%)
	till May 2024	1 (8.3%)
Median age in years	48.5 (17–76)	
Sex	Male	6 (50%)
	Female	6 (50%)
Smoker	Yes	2 (16.6%)
	No	10 (83.4%)
ASA grade	I	4 (33.3%)
	II	7 (58.3%)
	III	1 (8.3%)
Median weight in kilograms	56 (37–70)	
Median BMI in kg/m^2^	22.75 (16–29)	
Histology	Adenocarcinoma	3 (24.9%)
	Squamous cell ca	1 (8.3%)
	Mucoepidermoid Ca	2 (16.6%)
	Carcinoid	3 (24.9%)
	Ewings sarcoma	1 (8.3%)
	Metastatic OGS	1 (8.3%)
	Synovial sarcoma	1 (8.3%)
Laterality	Right	7 (58.3%)
	Left	5 (41.6%)
Neo-adjuvant therapy	Yes	3 (25%)
	No	9 (75%)
- **Preoperative treatment**: Neo-adjuvant therapy was given in the form of chemotherapy, Paclitaxel + Carboplatin for adenocarcinoma; EFT 2001 protocol (includes Vincristine, Dactinomycin, Adriamycin, Cyclophospamide, Ifosfamide and Etoposide) for primary lung Ewings sarcoma [9]; Methotrexate/Ifosfamide, Adriamycin and Cisplatin for Osteosarcoma (9,10).
- **Surgical details**: All the surgical procedures were performed by a single surgeon. Surgical procedures and approach are shown in Table 3.

**Table 3:** Surgical procedure and approach (*n* = 12)

**Surgical procedure**	
Left lower lobectomy	1 (8.33%)
Left lower lobectomy + SMLND	2 (16.66%)
Left upper lobectomy + sleeve bronchoplasty + SMLND	1 (8.33%)
Right lower bilobectomy + pericardial and diaphragm excision + SMLND	1 (8.33%)
Left upper lobectomy + SMLND + Chest wall resection (3–4 ribs)	1 (8.33%)
Right middle lobectomy	1 (8.33%)
Right upper bilobectomy + SMLND	2 (16.66%)
Right lower lobectomy + SMLND	2 (16.66%)
Right upper bilobectomy + SMLND + chest wall resection (3–6 ribs)	1 (8.33%)
**Surgical approach**	
VATS	3 (25%)
VATS converted to open	4 (33.3%)
Open	5 (41.7%)
  Broadly, the surgeries performed were 4-bilobectomies and 8-lobectomies. Chest wall resection was done for 2; for one patient, pericardial and diaphragm resection was done. For diaphragmatic excision, defect was closed with dual mesh. Surgical approach was decided based on size of the lesion, chest wall involvement and location of tumour. VATS procedure was converted to open in 4 patient. One had bleeding due to rent in pulmonary artery which could not be managed thoracoscopically and repair was done via open approach. Incomplete interlobar fissure was present in one and dense pleural adhesions in another patient. For one patient requiring sleeve bronchoplasty, anastomosis was performed via open approach.
- **Immediate post-operative complications: **The incidence of minor complications was 8.33%. Minor complications were SSI, that was managed with regular dressings and antibiotic as per culture. Major complications occurred in 4 (33.3%) patients. Two were major air leaks, defined as persistent air leak for more than 5 days requiring intervention with reinsertion of ICD or pigtail insertion. Two patients had collapse, managed by bronchoscopic toileting and aggressive chest physiotherapy. There was no 30-day mortality (Tables 4 and 5).

**Table 4:** Post-operative mortality and morbidity (*n* = 12)

Complication grade	Number of patients			Complication	Surgical procedure	Approach
Minor						
Grade I	0					
Grade II	1(8.33%)			SSI	Left lower lobectomy	VATS to open
Major						
Grade IIIa	4 33.3%	2	1	Major air leak	Left lower lobectomy	VATS to open
			1	Major air leak	Right lower lobectomy	VATS
		2	1	Collapse	Right upper bilobectomy + chest wall resection	Open
			1	Collapse	Left upper lobectomy + chest wall resection	Open
Grade IIIb	0					
Grade IVa	0					
Grade IVb	0					
Mortality						
Grade V	0

**Table 5:** Post-operative outcomes (*n* = 12)

Median time to chest drain removal	3 days (2–10)	
Median blood loss	450 mL (100–2000)	
Median hospital stay in days	Overall	5.3 (3–12)
	VATS	3 (3–8)
Final T stage	T1	1 (8.3%)
	T2	6 (50%)
	T3	2 (16.6%)
	T4	1 (8.3%)
N stage	N0	7 (58.3%)
	N1	3 (24.9%)
	Not applicable	2 (16.6%)
Margin	Positive	1 (8.3%)
	Negative	11 (91.6%)
Adjuvant	Yes	4 (33.3%)
	No	8 (66.6%)

The overall median duration of hospital stay was 5.3 days; for thoracoscopic procedure, it was 3 days. Adjuvant chemotherapy for non-small cell lung cancer was given for node positive cases and if tumour size was greater than 4 cm. Doxorubicin + Ifosfamide was given as adjuvant for synovial sarcoma.

## Discussion

The primary objective of this study was to analyse the introduction of thoracoscopic and open lung resections in a low-volume centre and to assess short term surgical outcomes. The study was conducted from September 2022 to May 2024, with a total of 12 patients who underwent pulmonary resections for primary and metastatic lung tumours. Our study results indicate that thoracoscopic and open lung resections can be effectively implemented even in a low-volume centre, with acceptable short-term outcomes.

### Post-operative Pulmonary Complications

In our study, post-operative pulmonary complications were major cause of post-operative morbidity. There was no 30-day mortality. Overall incidence of major pulmonary complications was 33.3%. Atelectasis/collapse and prolonged air leaks were the major grade IIIa complications. Atelectasis was seen in patients who underwent chest wall resections along with pulmonary resection. Minor complications grade I and II were 8.33%. SSI were minor complications. There were no grade IIIb or grade IV complications.

Advanced age is not a contraindication to curative resection in elderly [10]. However, advanced age is likely to lead to higher incidence of complications with coexisting comorbid conditions. Advanced age, smoking, comorbidities and complexity of surgical procedure increased the risk of developing PPCs. The rate of PPC marked significantly s were slightly higher when compared with other similar studies. The clinical and potential economic impact of PPCs is marked, with significantly longer hospital stay, ICU stay and number of deaths (Tables 6 and 7).

**Table 6:** Comparison with similar studies

Study	*n*	PPCs	30-day mortality
Stephen et al. [11]	266	25%	7.5%
Ayed et al. [12]	168	27%	2%
Jitesh et al. [13]	3045	28%	1.2%
Agostini et al. [14]	234	14.5%	2.1%
Mydral et al. [15]	616	8.8%	2.9%
Caushi et al. [16]	216	17%	2.3%
Gradica et al. [17]	968	4.9%	3.4%
Our study	12	33.33%	0%

**Table 7:** Factors for delivering safe outcomes after lung resections

Surgeon trained in thoracic surgery [18]
ERAS protocol [19]
Anaesthesia and critical care team trained in thoracic surgery [20]
Trained nursing staff [21]

Most frequent complication was prolonged air leak as previously reported by similar studies [11]. Prolonged air leak following lobectomy or wedge resection carries low mortality rate but is associated with prolonged hospital stay. Meticulous surgical technique and expertise can minimise air leaks. Atelectasis or collapse was also most frequent PPCs. Incidence of atelectasis can be minimised by smoking cessation, adequate post-operative pain control and intensive physiotherapy. VATS procedures were associated with reduced in-hospital complications, shorter hospital stay and lower need for post-operative analgesia [22, 23].

### Volume of Lung Resections

Many studies have shown that patients undergoing surgery for lung cancer benefit from receiving treatment in hospitals where high numbers of resections are carried out [24–27]. In contrast, a number of studies did not find an association between hospital volume and outcome after lung resections [28–31]. There is no consensus regarding definition of low-volume and high-volume centres for lung resections. Table 8 provides suggestions by different authors for defining high and low-volume centres for lung resections.

**Table 8:** Definition of low-volume and high-volume centres for lung resections

Author	Low volume	High volume
Harrison et al. [32]	< 40	> 40
Freixinet et al. [33]	< 43	> 54
Sioris et al. [34]	< 20	> 20
Lüchtenborg et al. [26]	< 70	> 150

Based on the above results, lung resections can be performed in a low-volume centre without compromising patient safety and quality of care with comparable short term results. We believe lung resections: both open and MIS scan be introduced to low volume centres safely in line with prior studies [21, 35].

### Pre-operative Care

Training of other personnel such as anaesthetists, operating nurses and nurses in the wards for ERAS fast track principles for improved perioperative outcomes is essential. Implementation of an ERAS protocol for pulmonary resection reduced length of hospital stay, morbidity and opioid use [19, 20]. We feel that presence of following factors can help in delivering safe outcomes after lung resections:

-ERAS protocol for pulmonary resections involves implementation of pre-operative-rehabilitation and nutritional support, smoking cessation
-Avoiding sedatives, VTE prophylaxis, maintenance of normothermia, euvolemia, lung protective strategies during single lung ventilation, regional anaesthesia to decrease post-operative opioid use, minimally invasive approach
-Post-operative- early removal of chest tube, considering digital drainage systems and suction when indicated, avoiding urinary catheters or early removal, mobilisation within 24 h of surgery and good physiotherapy helps in fast recovery and shorter hospital stay

### Experience at Homi Bhabha Cancer Hospital and Research Centre (HBCH&RC), Visakhapatnam

Ours is a tertiary cancer centre under the Tata Memorial Centre (Mumbai), Department of Atomic Energy, Government of India. At our centre, proper patient selection, pre-operative assessment and treatment planning by a multidisciplinary team is the mainstay. We have a dedicated Prehabilitation and Rehabilitation program that includes a team of physiotherapists, nutritionists and speech therapists. Managing challenges such as single-lung ventilation, intra-operative and post-operative ventilation, post-operative morbidity due to pulmonary complications and comorbidities requires team effort involving anaesthesiologists, surgeons trained in thoracic surgery and ICU nurses. We have achieved acceptable results at our centre due to this collaborative approach.

### Looking Forward

Future studies should investigate the long-term outcomes of thoracoscopic and open lung resections, including survival rates and quality of life from low-volume cancer centres. Additionally, there is a need for strategies to improve the surgical care for lung cancer patients in such low volume & resource constrained centres, such as training programs for surgeons and investment in surgical equipment.

Through the NCG (National Cancer Grid) and its member institutions, it is the right time to develop the cutoff for high-volume and low-volume centres for lung resections corresponding to our disease burden, resources and constraints. Also, uniform maintenance and reporting of outcomes after lung resections should be encouraged to create a national database under the NCG.

### Limitations

One of the main limitations of this study is the retrospective design, small sample size, and heterogeneity of performed operations. The data regarding potential predictors of perioperative outcomes such as pulmonary function tests was difficult to retrieve in some cases. Also, the study focused on short-term outcomes and complications, without assessing long-term factors like survival and quality of life. Future studies should consider these aspects for a more comprehensive understanding. Despite these limitations, our study provides valuable evidence supporting the feasibility of thoracoscopic lung resections in resource constrained settings. Further research is necessary to optimise patient outcomes and healthcare resources.

## Conclusion

Our study provides preliminary evidence that thoracoscopic and open lung resections can be successfully implemented in low-volume centres, with acceptable short-term outcomes. However, more research is needed to confirm these findings and to optimise patient care in such settings.
